# Determination of Biogenic Amine Level Variations upon Storage, in Chicken Breast Coated with Edible Protective Film

**DOI:** 10.3390/foods13070985

**Published:** 2024-03-23

**Authors:** Aneta Jastrzębska, Anna Kmieciak, Zuzanna Gralak, Kamil Brzuzy, Jacek Nowaczyk, Marcin Cichosz, Marek P. Krzemiński, Edward Szłyk

**Affiliations:** 1 Department of Analytical Chemistry and Applied Spectroscopy, Faculty of Chemistry, NCU in Toruń, 87-100 Toruń, Poland; 302301@stud.umk.pl (Z.G.); 302298@stud.umk.pl (K.B.); eszlyk@umk.pl (E.S.); 2 Department of Organic Chemistry, Faculty of Chemistry, NCU in Toruń, 87-100 Toruń, Poland; akmieciak@umk.pl (A.K.); mkrzem@umk.pl (M.P.K.); 3 Department of Physical Chemistry and Polymer Physical Chemistry, Faculty of Chemistry, NCU in Toruń, 87-100 Toruń, Poland; janowa@umk.pl; 4 Department of Chemical Technology, Faculty of Chemistry, NCU in Toruń, 87-100 Toruń, Poland; chemik@chem.umk.pl

**Keywords:** meat, meat coatings, chitosan, rosmarinic acid, biogenic amines

## Abstract

A new chitosan-based protective film containing rosemarinic acid (0.282% *w*/*w*) has been elaborated. The film was formed from a water–oil emulsion system and applied to poultry meat samples using a dip-coating technique. Various physicochemical parameters of the coatings, such as thickness, Young’s modulus, elongation at break, water vapor transmission rates, and antioxidant activity, were tested with free-standing film samples peeled from a Petri dish. Compared to neat chitosan films obtained similarly, new films cast from the emulsion showed significantly better elasticity (Young’s modulus was diminished from 1458 MPa to about 29 MPa). Additionally, barrier properties for moisture transition decreased from 7.3 to 5.8 g mm m^−2^ day^−1^ kPa^−1^. The coated poultry samples were subsequently evaluated in juxtaposition with uncoated ones in a storage test. Levels of selected biogenic amines (histamine, tyramine, tryptamine, phenylethylamine, putrescine, cadaverine, spermine, and spermidine), total bacterial count, and lipid oxidation levels in the meat samples were analyzed during storage at 4 °C (up to 96 h). The results obtained for the biogenic amines, total bacterial content, calculated biogenic amine index, and the ratio of spermidine to spermine in meat samples suggest the advantage of the proposed coatings with rosmarinic acid in protecting poultry meat against environmental factors and rapid spoilage.

## 1. Introduction

Meat and meat products constitute a good source of nutrients for the human diet because meat protein provides essential amino acids and is of high dietetic value. However, these products are prone to spoilage due to microbial activities, causing a risk to consumer health [1]. For example, fresh poultry products, such as chicken breast, are very convenient for the human diet due to their nutritional and sensory characteristics but have a short shelf life (2–4 days) caused by microbial contamination (psychrotrophic bacteria spoliation) and high water activity [2]. The meat quality is reduced by three main mechanisms: microbial degradation, lipid oxidation, and autolytic degradation in conjunction with enzymatic degradation. However, microbial spoilage is the main factor in this process [3,4]. This phenomenon causes protein degradation due to the progression and instigation of meat spoilage, producing contingent compounds that change meat: flavor, tenderness, juiciness, odor, texture, and appearance [5,6]. Among many metabolites formed during meat spoilage, particular attention should be paid to biogenic amines (BAs). Eight BAs, including spermidine (Spd), spermine (Spm), putrescine (Put), cadaverine (Cad), tryptamine (Trp), phenylethylamine (Phen), histamine (Him), and tyramine (Tyr) are most common in meat and meat products [7]. Among them, Him, Tyr, Cad, and Put are chemical indicators of meat spoilage, mainly due to the strong correlation with the bacterial counts in the meat samples. The formation rate of BAs depends on the availability of free amino acids, the activity of decarboxylase enzymes, and the environmental conditions (raw material, processing, and microbiota) [5]. It is well known that consuming food containing high concentrations of these compounds can cause adverse effects. According to Omer et al. [8], ingestion of large amounts of BAs in food causes toxicological effects and health disorders, including psychoactive, vasoactive, and hypertensive effects and respiratory, gastrointestinal, cardiovascular, and neurological disorders. Among BAs, tyramine and histamine are the ones most associated with food intoxication. For example, tyramine intoxication is associated with increased blood pressure, migraines, and hypertensive crises in individuals treated with monoamine oxidase inhibitors. The most common symptoms of histamine poisoning are due to the effects it has on the cardiovascular, gastrointestinal, and respiratory systems, producing low blood pressure, skin irritation, headaches, edemas, and rashes typical of allergic reactions. Putrescine and cadaverine enhance histamine toxicity and cause health problems such as abnormal blood pressure, allergic reactions to humans, headaches, and asthma tachycardia/worsening asthma [9,10].

Bioactive and eco-friendly food packaging is intended to extend the shelf life, quality, permanence, and presentation of meat to the customer. The bioactive coating of meat with a gelatin layer to delay microbe formation and loss of water was patented in the 19th century. Since then, the number of papers describing the possibility of using edible coatings has been constantly growing [4,5,11,12]. The increasing consumer preference for healthy and stable food and awareness of the harmful effects of synthetic packaging prompted research on new edible packaging materials or technology [13]. This innovative packaging also aims to improve the shelf life of meat by providing a barrier to moisture, gas, and lipids [14].

Additionally, edible coatings are applied directly to the food surface, creating a barrier that protects the product against environmental conditions. Potential benefits for the meat industry from edible coatings include preventing moisture loss, dripping juices, reducing oxidation of lipids and myoglobin, losing volatile flavor, and improving technological parameters [13]. The best thing about edible packaging for meat and meat products is that it stops pathogenic microorganisms from growing and going bad, and it also partially stops proteolytic enzymes from working on the surface of coated meat cuts [4].

Film-forming materials can be hydrophilic or hydrophobic; other components can be added to enhance their functional properties. Hydrocolloids (polysaccharides and proteins) and lipids are the most commonly used materials. Among these, polysaccharides are more suitable for producing films or coatings due to the presence of many hydroxyl groups susceptible to forming hydrogen bonds, prompting phase gelation [15]. Chitosan films (CSFs) have been extensively investigated for their ability to prolong the shelf life of various foods. This biopolymer has emerged as a promising material for antimicrobial applications in the food industry. Despite its relatively high price, chitosan has been proven to be biodegradable, nontoxic, and biocompatible, and can be isolated from abundant renewable natural sources [15]. Moreover, chitosan is generally recognized as safe (GRAS) by the U.S. Food and Drug Administration, European Union regulations, and China’s National Standards [16]. CFS’s antioxidant and antimicrobial activity against microorganisms such as Gram-positive and Gram-negative bacteria, filamentous fungi, and yeast were well documented and reviewed [6,15,16,17,18]. However, chitosan has some drawbacks, including low thermal stability, a low water vapor barrier, and ultraviolet decomposition, which limit its applications. These limitations could be minimized during the preparation of edible film [6,17].

The use of edible films for meat could be an option that provides both packaging and the necessary antioxidant and antimicrobial protection using natural and biodegradable components. Efforts are concentrated on creating the ideal combination of materials since the effectiveness of edible films for meat products depends on the types of biopolymers and bioactive compounds used to improve their functional properties [6].

A literature survey indicates that the main research area focuses on producing edible films with functional properties by incorporating antioxidants and antimicrobials into their formulation [2,19]. The possibility of introducing additives into the chitosan film that extend the shelf life of the meat makes it possible to improve the protective functions of the packaging. Edible films have been enriched with various antimicrobial agents, including organic acids, bacteriocins, essential oils, and extracts [17]. This kind of packaging combines the characteristics of environmentally friendly, zero-waste packaging with an innovative approach to active packaging. One of the research directions currently being developed is the introduction of functional components of rosemary (such as essential oil, oil, or oleoresin) into active packaging materials in different meat products [20]. For example, alternative food packaging has applied essential oils of rosemary [21,22,23], extracts, or dry herbs [24,25,26]. The antioxidant activity of rosemary products is attributed to phenolic diterpenes such as carnosol, carnosic acid, rosmanol, ursolic acid, and rosmaridiphenol, which can retard lipid oxidation by disrupting free radical chain reactions [20]. Furthermore, rosemary essential oil is accepted as a GRAS ingredient from plants.

Rosmarinic acid (RA) [27] is an outstanding example retarding the growth of microorganisms and inhibiting the increase in the pH value and oxidation in food. Several research studies mention using a 1% chitosan solution containing around 30 mg/L of RA [28] or equivalent herbal extract [29,30]. Although the results of these tests showed a significant increase in the durability of the food products tested [31], the deficiency of the coatings was their heterogeneous and fractured surface. For comparison, a pure chitosan coating has a smooth and compact surface. To address this issue, many changes to the composition of the film-forming solution were made as part of preliminary research before the studies described in this paper.

The research presented here looked into a new coating that can be used as edible packaging. It was based on the known method of enclosing rosmarinic acid in a chitosan polymer matrix. However, in our case, the solution was to dissolve RA in the oil phase, strengthening the chitosan film-forming structure.

Using an emulsion system instead of a conventional aqueous solution of chitosan has been based on the notion that various two-phase systems give rise to composite materials. These materials possess distinct properties that differ from the qualities of these systems’ individual components. In this case, the primary objective of employing a water/oil emulsion system was to establish a foundational structure to create an edible coating that can dissolve water-soluble and hydrophobic active compounds. Particularly considering the literature’s description of chitosan-based coatings that incorporate compounds with low water solubility, it is evident that these coatings exhibit unfavorable surface morphology [32]. The utilization of colloidal systems also adds complexity to the surface’s morphology. However, unlike fractures that are characteristic of the former, emulsion-based coatings exhibit a solid chitosan structure with trapped oil phase inclusions. This result could have been achieved by employing elevated concentrations of chitosan solution derived from an aqueous propionic acid solution, together with the incorporation of glycerin into the water phase and lecithin into the oil phase. The film-forming solution composition was developed through thorough preliminary research conducted before the current project, which is the focus of this article.

Another novelty was the use of this system to cover poultry meat. So far, examples regarding the protection of pork and fish [33] have been described in the literature. For this purpose, an innovative film-forming solution was developed to apply protective films directly to meat.

The coating was formed from a water/oil emulsion containing vegetable oils as a dispersive phase in an aqueous chitosan solution. Films obtained without (EF0.0) and with the addition of rosmarinic acid (EF0.r) were characterized by the chosen physicochemical parameters. The final stage of the work was to evaluate the impact of the edible coatings received on the formation of the total bacterial count, selected biogenic amines (Spd, Spm, Put, Cad, Trp, Phen, Him, and Tyr), and lipid oxidation levels in poultry meat during storage in the refrigerator for 96 h.

## 2. Materials and Methods

### 2.1. Reagents and Instruments

Analytical grade: cadaverine (Cad), putrescine (Put), spermine (Spm), spermidine (Spd), tyramine (Tyr), histamine (Him), tryptamine (Trp), phenylethylamine, 99% (Phen), rosmarinic acid (RA) 96%, 2,2′-azino-bis(3-ethylbenzothiazoline-6-sulfonic acid) diammonium salt (ABTS), 1,1,3,3-tetramethoxypropane, 2-thiobarbituric acid (TBA), 6-hydroxy-2,5,7,8-tetramethylchromane-2-carboxylic acid (Trolox), plate count agar (PCA) suitable for microbiology, NutriSelect^®^ Plus, Tryptic Soy Broth (TSB) proper for microbiology, NutriSelect^®^ Plus, L-α-lecithin, Soybean were purchased from Sigma Aldrich (Poznań, Poland). 2-Chloro-3-nitropyridine (3-CNP), potassium persulfate, barium chloride, sulfuric acid, butylhydroxytoluene (BHT), methanol (for HPLC), glycerol, perchloric acid, and trichloroacetic acid (TCA) were purchased from Alchem (Toruń, Poland). Chitosan powder (CS) was purchased from BioLogHoppe GmbH; the batch containing a degree of deacetylation of 98% and a dynamic viscosity of 1% soln. in 1% acetic acid was about 1200 mPss.

Meat samples were extracted using the Mars 6 microwave extraction system (CEM Corp., Matthews, CA, USA). Microwave derivatization reactions were performed with a Discover 2.0 microwave synthesizer (CEM Corp., USA). HPLC analyses were performed using a Shimadzu Corp. chromatograph (Kyoto, Japan) with an autosampler SIL-20AC HT and a photodiode multiwavelength detector (SPD-M20A Prominence Diode Array Detector). The LC solution program (1.23 SP version) was applied for chromatographic data processing. UV–Vis spectra were recorded using a Shimadzu UV1600 spectrophotometer in a 1 cm cell.

The film thickness obtained was determined using a constant load micrometer (Mitutoyo 547-526S). The films’ tensile strength and percentage elongation were determined using a Shimadzu texture testing machine (Shimadzu EZ-Test EZ-SX, Kyoto, Japan) with 3 cm jaw separation, a film width of 0.5 cm, and a strain rate of 0.5 cm min^−1^.

### 2.2. Preparation of Film-Forming Emulsions

The films studied were cast from emulsion complex mixtures obtained by emulsification of a particular portion of specific base solutions. Base solutions were prepared in the first step: 4% chitosan solution (CS), *w*/*w*, was obtained by adding 20 g of chitosan powder to 500 mL of 1% aqueous solution, *w*/*w* of acetic acid. The suspension was stirred using a mechanical stirrer (400 rpm) at room temperature for 48 h. The resulting viscous solution was filtered (sintered glass filter G1), and a clear solution was used for film formulations. The exact content of CS in the solution was determined gravimetrically by drying samples in the oven (70 °C) to the constant mass. The aqueous phase was obtained by mixing certain amounts of chitosan base solution with glycerol and water. The oil phase was prepared by mixing rice oil with lecithin, and with RA it was included in the formulation. Finally, the oil phase was poured into the aqueous phase, and the system was homogenized using a mechanical homogenizer at 3000 rpm for 20 min. The film-forming formulations were prepared, as specified in Table 1.

Free-standing films were developed by casting the emulsion into plastic Petri dishes, drying at 60 °C for 2 h, and then leaving for gelation in the fume hood under ambient conditions: 23 °C, 35% relative humidity (RH). After peeling off the Petri dish, the films were transferred to a conditioning box and kept at 25 °C for 24 h at 50% RH. Conditioned samples were subjected to further analysis.

### 2.3. Characteristics of the Edible Coatings

We tested chitosan coatings as stand-alone films to find out their basic mechanical strength parameters using the ISO standard for mechanical testing of plastic films [34] and their ability to keep water out using ISO/TS 21975:2020 [35] with some changes. The film thickness was determined, and the given values were the mean of 10 measurements after equilibration at 25 °C and 60% RH. The standard gravimetric method measures water vapor transmission rates (WVTR) through the films. Four replicates of each sample type with 10 cm^2^ of exposed area were mounted on the test vessel filled with distilled water. The RH inside the container was 80% (pH_2_O = 2.54 kPa). Containers were placed inside the desiccator (dry CaCl_2_) at RH = 28% (pH_2_O = 0.89 kPa). The mass of the testing vessels was measured twice a day for one week. The WVTR values were calculated by applying a least-squares analysis. According to standard comparison, different specimens are reasonable after the standardization procedure, considering the dimensions of the individual samples. In this case, the method necessitates dividing the area of the exposed barrier by its thickness. Consequently, we obtained the water vapor permeability (WVP) expressed in g·mm·m^−2^ kPa 9 [34].

The antioxidant activity of the coatings obtained (AA) was determined using the ABTS [2,2′-azino-bis(3-ethylbenzothiazoline-6-sulfonic acid) diammonium salt] assay. A stock of radical cation ABTS (ABTS^•+^) was prepared by a reaction of 7 mM ABTS solution with 2.45 mM potassium persulfate and incubated in the dark at 25 °C for 24 h. Next, 0.1 mL of ABTS^•+^ solution was mixed with different masses (about 20 mg) of the coating obtained and made up to 10 mL by ethanol. The absorption was measured after 15 min at 734 nm against ethanol as a blank. The AA of the tested films was expressed as micromoles of Trolox equivalents per 1 g of edible coating according to the calibration curve: %ABTS = (4 × 10^6^) C + 14.659 (R^2^ = 0.999).

### 2.4. Preparation of Chicken Breast Coated with Chitosan Films

Fresh chicken breasts (weight: 1.5 kg) were purchased from a local market and transported to the laboratory in less than 30 min. Before the experiments, the meat was not subjected to any thermal processing (freezing or thawing). Fresh meat was cut aseptically into 20 g pieces (length: 10 cm) and completely dipped for 5 min at room temperature in test films. Subsequently, meat samples coated with edible film were left for 30 min to remove excess formulation and air-dried. The samples were coated with an emulsion containing rosmarinic acid RA (code M—EF0.r) and without RA (code M—EF0.0). As described above, a coatless control meat sample (CM code) was also prepared. All samples were placed in an airtight polypropylene container and refrigerated at 4 °C for 96 h. Sampling was conducted at 24, 48, 72, and 96 h of storage. Both chitosan films (EF0-0 and EF0-r) were prepared three times in separate beakers. Experiments were conducted on three samples of meat dipped in the resulting solutions.

### 2.5. Total Bacterial Count (TBC) in Meat Samples

Microbial counts were carried out immediately after transporting meat samples to the laboratory before other analyses. Total visible bacteria counts were carried out according to ISO 4832:2006 [36] and ISO 4833-1:2013 [37] after incubation at 30 °C for 72 h. Five to six grams of meat samples were weighed in a sterile stomacher bag filter (Interscience, Saint Nom la Brétèche, France) with 0.1% sterile peptone water (about 100 g). The samples were mixed for three minutes using a stomacher (BagMixer 400 P, Interscience, Saint Nom la Brétèche, France). The homogenized solution was the stock solution (dilution 10^−1^). The first 10^−1^ dilution (1 mL was transferred to 9 mL of peptone water) and serial decimal dilutions up to 10^−6^ were prepared as described by Mohammed et al. [38]. The total bacterial count (TBC) was determined on plate count agar (PCA, BIOCORP Poland) after incubation at 30 °C for 72 h. The colonies developed on plates were counted using the cellSence Dimension software version 1.15 (EVIDENT (OLYMPUS), Tokyo, Japan). Antibacterial inhibition was determined by the colony counting method, and the obtained results were presented as log_10_ CFU·g^−1^. Each experiment was carried out in triplicate and presented as the mean ± standard deviation.

### 2.6. The Lipid Oxidation Level Determination (TBARS Test)

The level of 2-thiobarbituric acid reactive substances (TBARS) was measured using the method described by Salih et al. [39]. The samples of tested meat (10.0 g) were homogenized with a mixture of 4% perchloric acid and 0.01% butylhydroxytoluene (BHT) solution for 2 min, centrifuged at 9000 rpm for 15 min, filtrated, and diluted with 4% perchloric acid in a volumetric flask (50 mL). Next, 5 mL of the obtained solution was diluted with 0.02 M TBA solution to 10 mL and heated at 90 °C for 60 min. After cooling, the TBARS present in the supernatant was determined by measuring the absorbances at 532 nm. TBARS expressed as mg·kg^−1^ was calculated according to the calibration curve for 1,1,3,3-tetramethoxypropane. The analyses were performed fivefold and presented as the mean ± standard deviation.

### 2.7. Biogenic Amine (BA) Analysis with the HPLC Technique

The levels of the selected BAs (Him, Tyr, Trypt, Phen, Put, Cad, Spd, and Spm) were examined immediately after transferring the meat to the laboratory and on each day of storage. The control and coated meat samples were homogenized in a commercial blender, and the selected BAs were determined according to the procedure described before [40]. In the first stage, microwave extraction was performed with 5% trichloroacetic acid (TCA), followed by microwave derivatization with 2-chloro-3-nitropyridine (150 °C, 20 min). The obtained derivatives of BAs were analyzed using RP-HPLC. Analyses were carried out using a Gemini 5 µm NX-C18 LC column 250 × 4.6 (Phenomenex LTD., Torrance, CA, USA). The mobile phase was methanol (solvent A) and water (solvent B), and the gradient conditions were: 0 min A: 60%; 0–40 min A: 100%; 40–45 min A: 60%. Separation was carried out under the following conditions: total flow rate—1 mL·min^−1^; temperature—35 °C; injection volume—20 µL, and detection wavelength—360 nm [40]. Calibration curves were prepared on the same day using working solutions of BAs derivatives in methanol. The least-squares method was applied to calculate the line’s equations, resulting in determination coefficients between 0.9952 and 0.9999.

Five samples were prepared and analyzed in triplicate each time, presenting the results as mean ± standard deviation. Data were analyzed by a two-way ANOVA, and Tukey’s multiple range tests (*p* < 0.05) were used to determine the significance between the various treatments.

## 3. Results and Discussion

When it comes to the design of food packaging, including edible packaging, having a solid understanding of the mechanical properties of materials is highly important. Flexibility, strength, and elasticity are the factors that govern how a material reacts to the application of physical forces during handling, transit, and use. Finding the best balance between rigidity and flexibility is a substantial challenge.

The literature data indicate chitosan as a material with poor mechanical and barrier properties [41]. The presented study examined an innovative solution of casting chitosan films from a water/vegetable oil emulsion as a dispersive phase. These films are edible coatings made of exclusively edible natural ingredients. The oil phase was applied as a mechanical reinforcer and a carrier of hydrophobic additives in the tested systems. The physical and mechanical properties of the tested coatings in the form of free-standing films were compared to similar films made of pure chitosan. The appropriate tests were conducted to assess the impact of two-phase structures brought about by the colloidal nature of the film-forming fluid on stiffness, tensile strength, and water vapor permeability.

The results of these tests are reported in Table 2 and presented as mean ± standard deviation.

Considering the data collected in Table 2, it can be concluded that the film parameters obtained from the emulsions differ significantly from the pure chitosan solutions. The CSF foil is characterized by high stiffness (Young’s modulus value over 1000 MPa) and is very brittle, reflecting low elongation at break. On the other hand, the films obtained from the emulsion are flexible, Young’s modulus is two orders of magnitude lower, and their elongation at break is higher than that of CSF. Both differences reflect the high elasticity of the material. It should also be noted that rosmarinic acid does not cause significant changes in the mechanical parameters of the respective films (EF0.0 and EF0.r). The barrier properties of the tested films do not differ significantly from typical examples of chitosan-based films. It was also evident that the water-vapor permeation coefficient (WVP) is lower for systems obtained from the emulsion composition than for pure chitosan. The latter indicates that films obtained from emulsion have better barrier properties than pristine chitosan films. This is probably due to a dispersed oil phase in the matrix of the hydrophilic chitosan hydrogel. The additional phase, impenetrable for water, partially blocks the effective surface available for water diffusion but increases the tortuosity factor of diffusion.

In this paper, we proposed rosmarinic acid (RA) as a compound to improve the properties of the developed film. Despite the lack of differences in the mechanical properties of the coatings obtained, even a small addition of RA (<0.3% *w*/*w*) has influenced the antioxidant activity of the discussed packaging. The determined results of the ABTS procedure expressed as µM of Trolox equivalent per 1 g of coating were as follows: 3.81 ± 0.15 µM·g^−1^ for EF0.r, while 1.89 ± 0.06 µM·g^−1^ for EF0.0. According to the literature [42], chitosan exhibits antioxidant activity. However, this property of chitosan is directly proportional to its molecular weight, concentration, and viscosity. Moreover, the degree of deacetylation determines the scavenging capacity of chitosan, and the NH_2_ groups are responsible for the free radical scavenging effect. Nonetheless, the antioxidant efficacy of chitosan is constrained by the lack of an H-atom donor, which is necessary to serve as a good chain-breaking antioxidant. Additionally, the hydroxyl and amino functional groups exhibit limited reactivity with hydroxyl radicals, attributable to the robust intramolecular and intermolecular hydrogen bonding that impedes their dissociation. Whereas rosemarinic acid is considered the most potent antioxidant of all hydroxycinnamic acid derivatives, several studies have shown this polyphenolic compound’s biological and protective efficacies, including antibacterial and antioxidant activities [27]. Despite the well-described antioxidant potential, RA is characterized by poor bioavailability due to high instability, inefficient permeability through biological barriers, and poor water solubility [43]. As observed in this study, the pronounced increase in the antioxidant activity of the film with rosmarinic acid probably resulted from the synergistic action of chitosan and rosmarinic acid.

**Figure 1:**
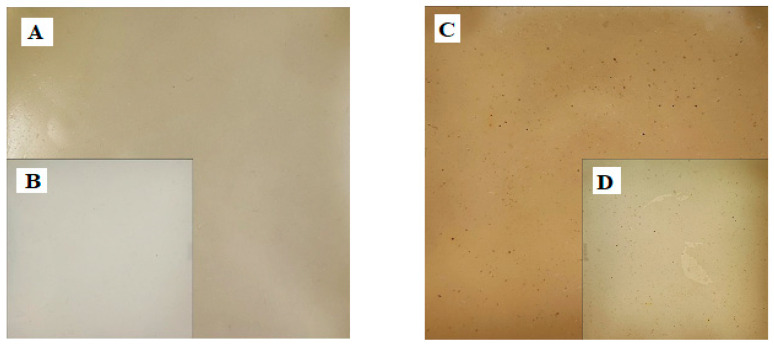
Photo of received coating—EF0.0 (**A**,**B**) and EF0.r (**C**,**D**), where (**B**,**D**)—photo obtained using LED Photo Shadowless Light.

**Figure 2:**
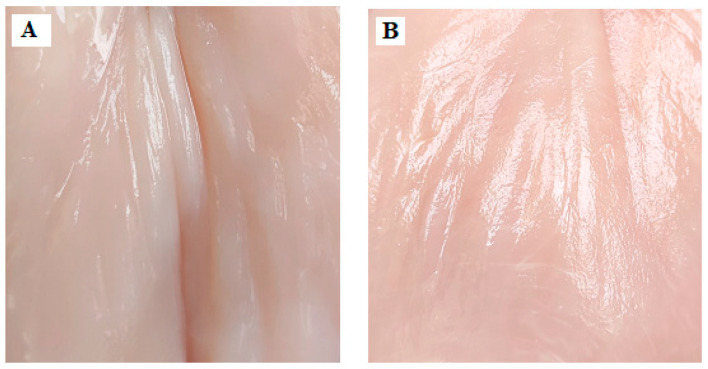
The photo of control meat samples: CM (**A**) and M-EF0.r (**B**) after 48 h of storage at 4 °C.

It should be added that the colors of the elaborate coatings are different, but the light and transparent EF0.0 (Figure 1A,B) and the darker EF0.r (Figure 1C,D) did not affect the coloration of the meat (Figure 2A,B). The latter confirms that the proposed coatings used as meat packaging will not alter the visual perception of the meat.

Literature data on potential applications of RA as a natural antioxidant in food coatings indicate the growing interest of research groups. It should be noted that Li et al. [28] proposed chitosan-based coatings with RA and ε-polylysine for fish fillets. Others, like Ge et al. [44], introduced novel edible films based on active gelatin with rosmarinic acid and discussed their properties, such as water resistance properties, mechanical properties, light barrier capacity, and antioxidant and antibacterial activities. In both cases, prepared edible films revealed promising features important for applications in food packaging.

### Results of Selected Parameter Determination in Meat Samples

**Figure 3:**
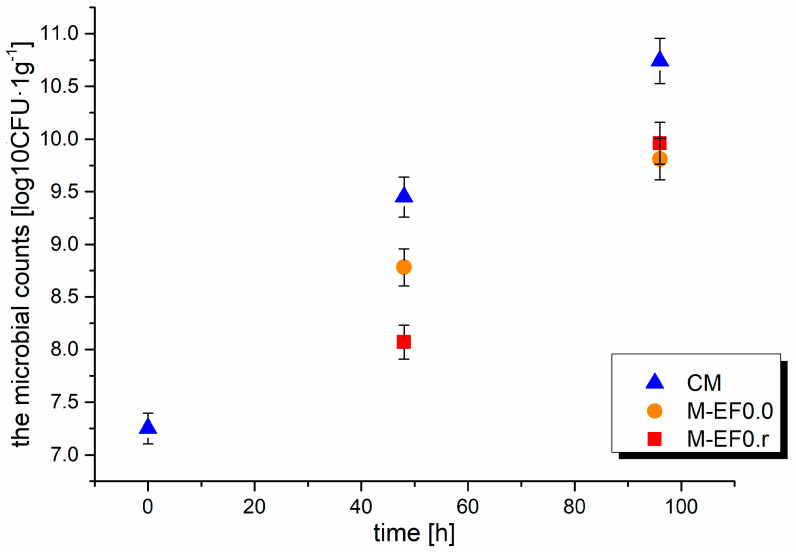
Results of the microbial counts [log_10_CFU·g^−1^] in meat after 48 and 96 h of storage at 4 °C; where: CM—control meat, M-EF0.0—meat samples with coating, M-EF0.r—meat samples with coating and RA.

**Figure 4:**
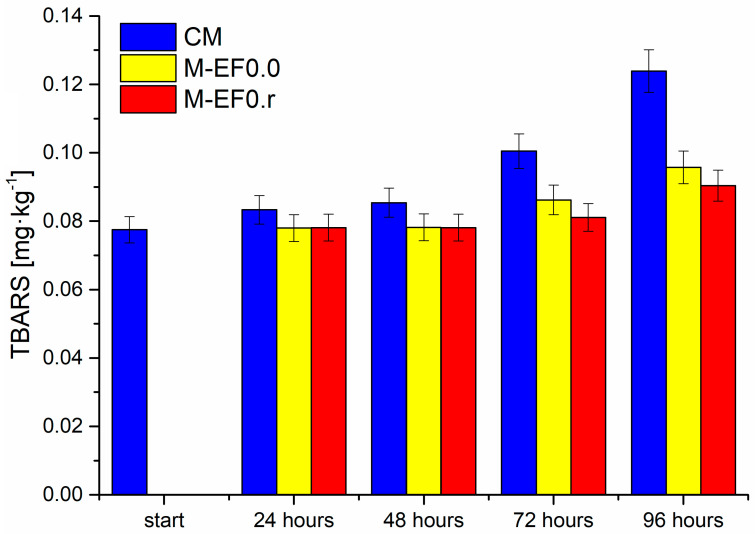
Results of TBARS contents [mg·kg^−1^] in meat samples during storage at 4 °C, where: CM—control meat, M-EF0.0—meat samples with coating, M-EF0.r—meat samples with coating and RA.

**Figure 5:**
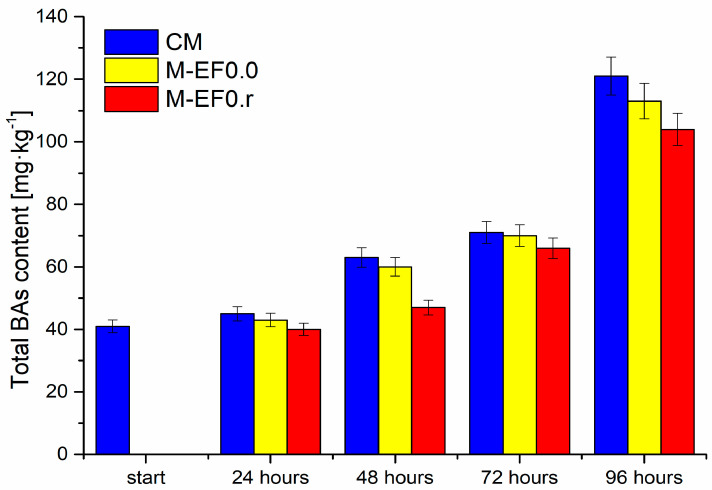
The total BAs content [mg·kg^−1^] in meat samples during storage at 4 °C, where: CM—control meat, M-EF0.0—meat samples with coating, M-EF0.r—meat samples with coating and RA.

According to Song et al. [6], coating food with chitosan films lowers the partial oxygen pressure in the package, maintains temperature with moisture transfer between the food and its environment, controls respiration, and decreases dehydration. Adding active compounds, such as rosmarinic acid, to edible coatings is not a novel technique, but it expands the versatility and utility of edible films [4]. This work examined the impact of edible chitosan coating with rosmarinic acid on the TBC, TBARS, and BAs content in poultry meat (chicken breast) during storage. The results were compared with the coating without RA and raw meat. Total bacterial, TBARS, and biogenic amine content are presented in Figure 3, Figure 4 and Figure 5, whereas obtained results (as mean ± standard deviation) are listed in Tables S1 and S2 (Supporting Materials).

Meat is a good support for bacterial growth, resulting in microorganisms’ impact on the storage life of meat products because meat is composed of 75% water and many different bioorganic molecules such as amino acids, peptides, nucleotides, and sugars. The presence of bacteria on the surface of meat depends on the environmental conditions (temperature, oxygen availability, water activity) and the initial microbiota [45]. Furthermore, chicken muscle tissues contain monounsaturated and polyunsaturated fatty acids, which O_2_ rapidly oxidizes in the presence of light. Usually, meat stored in the air is quickly infected by bacteria, which was observed during our experiment. The changes in the aerobic bacteria population during meat storage are summarized in Figure 3. For control meat sampled at 48 and 96 h, the total plate count of microbes increased sharply from 7.25 log_10_CFU·g^−1^ to 9.45 log_10_CFU·g^−1^ and 10.74 log_10_CFU·g^−1^. A better effect of inhibiting the microbiota development after 48 h of storage was observed for M-EF0.r; moreover, the values obtained: 8.78 log_10_CFU·g^−1^ for M-EF0.0 and 8.07 log_10_CFU·g^−1^ for M-EF0.r differed statistically significantly (Anova, Tuckey test). The lower TBC values observed for M-EF0.r can probably be caused by the addition of RA, which affected the bactericidal properties of the coating.

The literature data [46] indicate that chitosan in the films exhibits an antimicrobial effect on Gram-negative and Gram-positive bacteria. However, due to the nonidentical composition of cell walls, the interaction of chitosan differs from that of these bacteria. Numerous hypotheses have been proposed regarding the antibacterial mechanisms of chitosan and its derivatives; however, the precise mode of action remains incompletely elucidated. Moreover, many factors can influence the antibacterial activity of chitosan [47]. The antimicrobial activity of chitosan is believed to occur when the compounds adhere to the surface of bacterial cells. This adherence is followed by increased permeability of the cell’s lipid membrane, causing essential compounds to exit the cell, leading to cell death [48]. Another possible mechanism is that chitosan acts as a chelating agent that selectively binds to trace metal elements, causing toxin production and inhibiting microbial growth. However, regardless of the likely mechanism of action, the polycationic structure of chitosan is a prerequisite for its antibacterial activity [49]. In the case of RA, its antimicrobial activity has been discussed in many studies [27]. This polyphenol exerted antimicrobial effects against *Enterobacteriaceae* spp., *Pseudomonas* spp., lactic acid bacteria, yeast and mold, and psychotropic counts, as well as fate *Listeria monocytogenes* inculcated in chicken meats [21,27]. Moreover, RA also displayed inhibitory effects against the *Staphylococcus aureus* cocktail by inducing morphological changes, reducing viable cell counts, and causing morphological changes in cheese and meat samples [27].

Microbial contamination of meat during storage had several implications, such as fouling and softening of muscular tissue and increasing BA levels (Figure 5), consistent with the literature data [50].

The next parameter examined in the discussed poultry meat was the 2-thiobarbituric acid reactive substances index (TBARS). The test applied in this study is one of the classical methods for assessing lipid oxidation in meat and meat products, where the secondary oxidation products of the lipids are reacted with 2-thiobarbituric acid [51,52]. As presented in Figure 4, the discussed index in all tested meat samples increased during the storage time. However, the degree of lipid oxidation was highest for meat not protected by any coating. The value of TBARS for these samples increased significantly from 0.0785 mg·kg^−1^ to 0.1239 mg·kg^−1^ after 96 h of storage. For M-EF0.0 and M-EF0.r samples, the level of TBARS was practically the same after 24 and 48 h of storage, at approximately 0.081 mg·kg^−1^. After 72 and 96 h of meat storage, we observed higher TBARS values for M-EF0.0; however, the differences between the two samples (M-EF0.0 and M-EF0.r) were not statistically significant (Tukey’s multiple range tests, *p* < 0.05). Considering the obtained values of antioxidant activity for both coatings (3.81 µM TE·g^−1^ for EF0.r and 1.89 µM TE·g^−1^ for EF0.0), it was surprising for us. Different factors could contribute to the increased TBARS values while storing tested samples. As mentioned above, the antioxidant nature of chitosan and rosemarinic acid is well documented.

Furthermore, the synergistic effect of these two antibacterial agents is described in the literature [46,49]. However, the results suggest that the restriction of lipid oxidation should instead be associated with the inhibition of oxygen diffusion to the meat surface rather than with the antioxidant activity of RA. It can be concluded that TBARS index formation was depressed by both packagings, although the presence of RA with emulsion did not cause significant differences.

The high protein levels in poultry meat contribute to proteolysis and increase autolysis, affecting amino acids release. The latter can be coupled with bacteria capable of decarboxylation, resulting in meat spoilage acceleration, and an increase in the content of microbial metabolites, including biogenic amines, is observed. Figure 5 illustrates changes in the content of all determined biogenic amines and BAs (calculated as the sum of BAs) in the tested poultry meat. The most significant increase in the total quantity of BAs was observed between 72 and 96 h of storage, while the smallest increase occurred after 24 h and between 48 and 72 h, respectively. Moreover, it can be observed that their quantity depends on the meat coating applied. The lowest increases in the content of the discussed compounds were observed for M-EF0.r.

The Pearson correlation coefficient was applied to measure a linear correlation between two variables, and the obtained results are presented in Table 3.

All tested parameters (TBARS, sum of all BAs, and TBC) correlated positively, and the calculated Pearson correlation coefficient varied from 0.8242 to 0.9990. Surprisingly, the highest positive correlations were obtained between the examined parameters for the M-EF0.r samples.

The results of the individual BAs’ determination in meat samples and selected calculated indexes (BAI and Spd/Spm) are listed in Table 4.

The fresh meat contained only two naturally occurring polyamines: spermidine (Spd) and spermine (Spm), with a predominant spermine content. The considerable variation in the amines content was noted in the literature. Furthermore, the levels of polyamines observed during the storage of poultry meat may vary with storage time, meat species, and type of packaging [53]. The ratio between spermidine and spermine (Spd/Spm) is considered one of the most important indices for evaluating chicken meat quality because it is independent of the type of flora. According to the literature [54], the levels of Spm and Spd remain almost constant or decrease slightly during meat storage. A similar situation was observed in our study. During storage, the concentration of these polyamines decreased marginally but consistently in all meat samples evaluated. Still, the application of each coating significantly slows down the degradation process. This is particularly evident in the case of Spd; the level in meat decreased by 73% during 96 h of storage, while in film-protected meat, the decrease was 29% for M-EF0.0 and 9% for M-EF0.r, respectively.

The decrease in the Spm content is likely associated with the enzymatic reaction of polyamine oxidases or caused by microorganisms that used Spm as a nitrogen source. Based on the Spd/Spm ratio, it is evident that during 24 h of storage, the value was practically constant for all meat samples. The Spd/Spm ratio remained unchanged for M-EF0.0 and M-EF0.r after 48 h, compared to CM. The latter suggests that both coatings protected the meat from unfavorable environmental conditions during 48 h. However, after this time, there were significant differences between M-EF0.0 and M-EF0.r (Anova, Tukey test), indicating better meat protection by EF0.r (Table 4). The aged meat microbiota may contain lactic acid bacteria (LAB), and mesophilic and psychrotrophic bacteria. Additionally, bacteria from the genera *Bacillus*, *Clostridium*, *Pseudomonas*, *Photobacterium*, *Citrobacter*, *Escherichia*, *Proteus*, *Micrococcus*, and *Lactobacillus*, which appear in meat during the aging process, can produce decarboxylases that lead to the formation of BAs [7]. On the other hand, as mentioned above, these polyamines occur naturally in meat, and their quantity is independent of the microbiota responsible for the formation of biogenic amines. Moreover, some spoilage-responsible microorganisms might have a different biogenic amine-forming capacity.

Therefore, we suggest that the difference in the Spd/Spm ratio for meat samples (M-EF0.0 and M-EF0-r) after 48 h of storage could be the result of the synergistic interaction of chitosan and RA on the antibacterial properties of the applied films.

A significant increase in the total amount of BAs after 48 h (Figure 5), discussed above, was observed from the rise in the cadaverine level (Table 4), which was also noticed by Wojnowski et al. [55]. Cadaverine (Cad), histamine (Him), putrescine (Put), and tyramine (Tyr) are chemical indicators of meat spoilage due to their strong correlation with bacterial counts. In the case of Cad, its concentration in meat is determined by microbial enzymatic activities and characteristics of the meat tissue. In our study, a significant increase in Cad levels was observed, most likely due to the rapidly increasing number of bacteria in the meat (Figure 3). Furthermore, in the case of poultry meat, spoilage occurs earlier than in other types of meat due to the presence of shorter protein chains, leading to the faster generation of amino acids which are precursors of BAs [53].

It should be noted that, for M-EF0.0 and M-EF0.r, an increased Cad level was also observed after 48 h; even so, it was not as high as for CM. The content of the Put (second physiological diamine) increased in all meat samples during the 48 and 96 h of study. The highest level of Put was observed for CM, whereas the lowest and slowest growth was noticed for M-EF0.r. Both diamines, Put and Cad, are identified as toxic BAs, as they favor intestinal absorption of Him and Tyr and contribute to a reduction in catabolism, thus enhancing their toxicity [53]. These four biogenic amines are included in the BAI index, and, according to the observed value, the CM sample was qualified as low quality after 48 h, and the meat was spoilt after 96 h of storage [50].

It is worth mentioning that chitosan-based films are resistant to fat, oil, and oxygen but highly sensitive to moisture, which probably caused the increase in the total content of BAs and the BAI index after 48 h of storage for M-EF0.0 (Figure 5 and Table 4), and in 72 h for M-EF0.r.

An intake of 750 to 900 ppm of BAs has been advised as a maximum limit; >1000 ppm is deemed a health hazard. International permissible limits of biogenic amine consumption in meat and meat products are absent. However, some countries (Netherlands, Czech Republic) proposed a permitted limit of histamine in meat products below 200 ppm [8]. On the other hand, the maximum level of tolerated Him content in meat was determined as 100 mg·kg^−1^, whereas daily consumption should not exceed 50 mg for histamine and 600 mg for tyramine [50]. The results obtained for the histamine, tyramine, and total biogenic amine content are below the abovementioned limits. It should be emphasized that the toxicity thresholds of BAs in food are not established, as they vary based on an individual’s gastrointestinal system’s detoxification capacity for each compound under discussion. The latter illustrates the importance of research on assessing the content of these compounds and developing methods for their reduction in food.

Analyzing the total and individual content of biogenic amines, the calculated BAI index, and the Spd/Spm ratio, it can be seen that the EF0.r film evokes a significant inhibition of these compounds’ content in meat. It is essential to note the impact of RA on the edible film on the content of Tyr and Him in poultry meat. These toxic BAs were not detected after 48 h of storage, while after 96 h, their levels were similar to those found in M-EF0.0. Chitosan is known to have antioxidant potential with some limitations; however, this parameter of chitosan films has increased after doping with natural antioxidants. The latter may cause a significant decrease in BAs levels in M-EF0.r.

One of the advantages of EF0.r films was the inhibition of Him presence in meat and the reduction of Put and Cad content. The poultry meat protected in this way retains its organoleptic qualities for 48 h of storage. Therefore, the proposed coating can be used as antimicrobial poultry meat packaging material, as it protects the meat from undesirable environmental factors and reduces microbial contamination on the surface of the packaged products.

## 4. Conclusions

The spoilage of chicken meat during storage usually causes safety concerns and economic losses. One way to improve poultry meat’s quality and microbiological safety is to cover it with an edible coating. In this study, chitosan-based coatings containing rosemarinic acid encapsulated in oil-phase microdroplets were developed and tested. The obtained results of selected physicochemical parameters confirm significantly better elasticity and barrier properties for moisture transition in comparison to the neat chitosan films obtained in the same manner. Finally, the discussed chitosan film was applied to poultry meat samples using dip-coating. Resulting specimens were applied to coated poultry samples which were subsequently evaluated in juxtaposition with the uncoated ones in a storage test.

Although the results are not strongly conclusive, promising effects have been observed in our experimental conditions. The level of all biogenic amines was reduced in meat coated with the proposed film comparing the raw meat sample. Furthermore, similar conclusions can be suggested for the other investigated meat quality parameters (TBARS and TBC). Based on the obtained results we can suggest that the new edible multiphase coatings with rosmarinic acid may improve poultry meat quality when stored in household refrigerators. However, further research is necessary to optimize the minimum level of rosmarinic acid in coatings that would inhibit the development of microorganisms responsible for generating BAs in meat.

## Figures and Tables

**Table 1:** Formulations of film-forming emulsions.

Code for Coating	CS (%, *w*/*w*)	Glycerol (%, *w*/*w*)	EO (%, *w*/*w*)	Lecithin (%, *w*/*w*)	RA (%, *w*/*w*)
CSF	2.000	0.000	0.000	0.000	0.000
EF0.0	2.000	0.158	0.184	0.002	0.000
EF0.r	2.000	0.162	0.191	0.002	0.282

Where: CSF—reference chitosan coating; EF0.0—coating without rosmarinic acid; EF0.r—coating with rosmarinic acid; CS—chitosan; EO—edible oil; RA—rosmarinic acid.

**Table 2:** Physico-mechanical parameters of studied coatings in the form of free-standing films.

Property	Units	CSF	EF0.0	EF0.r
Thickness	×10^−6^ m	187 ± 2	295 ± 3	312 ± 5
Young’s Modulus	MPa	1458 ± 28	28.7 ± 1.3	29.3 ± 1.5
Elongation at break	%	5.7 ± 1.5	22.5 ± 2.3	20.3 ±1.9
WVP	g mm m^−2^ day^−1^ kPa^−1^	7.3 ± 0.2	5.8 ± 0.2	5.6 ± 0.3

Where: WVP—water-vapor permeation coefficient; CSF—reference chitosan coating; EF0.0—coating without rosmarinic acid; EF0.r—coating with rosmarinic acid.

**Table 3:** The calculation of Pearson correlation coefficients.

	CM		M-EF0.0		M-EF0.r	
	TBC	TBARS	TBC	TBARS	TBC	TBARS
TBC						
TBARS	0.8712		0.8242		0.9668	
SBAs	0.9196	0.9940	0.9292	0.9751	0.9774	0.9990

Where: TBC—total microbial counts, TBARS—2-thiobarbituric acid reactive substances index, SBAs—calculated sum of all BAs.

**Table 4:** Results of the determination of BAs (average value ± standard deviation) [mg·kg^−1^] in meat samples during storage at 4 °C.

Sample	Him	Tyr	Trp	Phen	Put	Cad	Spd	Spm	BAI	Spd/Spm
CM	nd	nd	nd	nd	nd	nd	8.99 ^A^ ± 0.32	31.79 ^A^ ± 1.92	0	0.28 ± 0.02
24 h										
CM	2.88 ^I^ ± 0.18	2.47 ^F^ ± 0.18	nd	nd	nd	0.33 ^J^ ± 0.03	8.85 ^A,B^ ± 0.11	30.47 ^C,F^ ± 0.96	5.69 ± 0.25	0.29 ± 0.01
M-EF0.0	2.69 ^J^ ± 0.12	nd	nd	nd	nd	nd	8.85 ^A,B^ ± 0.17	31.25 ^A,C^ ± 0.31	2.70 ± 0.12	0.28 ± 0.01
M-EF0.r	nd	nd	nd	nd	nd	nd	8.95 ^A^ ± 0.19	31.44 ^A,B^ ± 0.42	0	0.28 ± 0.01
48 h										
CM	5.26 ^F^ ± 0.14	2.71 ^D,E^ ± 0.16	nd	nd	3.59 ^G^ ± 0.08	14.80 ^F^ ± 0.13	6.45 ^G^ ± 0.11	29.97 ^D,E,F,G^ ± 0.48	26.43 ± 0.21	0.21 ± 0.01
M-EF0.0	4.12 ^H^ ± 0.11	2.60 ^E^ ± 0.08	nd	nd	3.02 ^H^ ± 0.10	10.80 ^H^ ± 0.12	8.75 ^B,C^ ± 0.16	30.70 ^B,C,D^ ± 0.14	20.54 ± 0.18	0.28 ± 0.01
M-EF0.r	nd	nd	nd	nd	0.95 ^I^ ± 0.08	5.93 ^I^ ± 0.09	8.84 ^A,C,D^ ± 0.12	30.96 ^A,C^ ± 0.21	6.88 ± 0.14	0.28 ± 0.01
72 h										
CM	9.51 ^B^ ± 0.10	2.78 ^D^ ± 0.12	nd	nd	7.08 ^D^ ± 0.11	17.80 ^E^ ± 0.13	5.61 ^H^ ± 0.09	28.52 ^I^ ± 0.09	37.10 ± 0.24	0.20 ± 0.01
M-EF0.0	5.87 ^E^ ± 0.22	2.64 ^E^ ± 0.10	nd	nd	5.15 ^E^ ± 0.07	20.77 ^D^ ± 0.16	6.91 ^F^ ± 0.12	29.66 ^F,H^ ± 0.09	34.43 ± 0.26	0.23 ± 0.01
M-EF0.r	4.97 ^G^ ± 0.11	2.60 ^E,F^ ± 0.14	nd	nd	4.68 ^F^ ± 0.11	14.40 ^G^ ± 0.15	8.70 ^B,D^ ± 0.14	30.52 ^C,E^ ± 0.22	26.65 ± 0.33	0.28 ± 0.01
96 h										
CM	10.8 ^A^ ± 0.12	5.48 ^A^ ± 0.06	15.30 ^A^ ± 0.09	7.20 ^C^ ± 0.07	27.01 ^A^ ± 0.10	27.40 ^A^ ± 0.10	2.45 ^I^ ± 0.10	25.38 ^J^ ± 0.11	70.68 ± 0.19	0.10 ± 0.01
M-EF0.0	7.93 ^C^ ± 0.13	5.35 ^B^ ± 0.05	14.51 ^C^ ± 0.12	8.07 ^A^ ± 0.08	14.70 ^B^ ± 0.12	27.18 ^B^ ± 0.29	6.39 ^G^ ± 0.08	28.59 ^I^ ± 0.80	55.17 ± 0.38	0.22 ± 0.01
M-EF0.r	7.66 ^D^ ± 0.09	4.97 ^C^ ± 0.09	14.80 ^B^ ± 0.11	7.93 ^B^ ± 0.10	8.12 ^C^ ± 0.08	22.91 ^C^ ± 0.14	8.15 ^E^ ± 0.07	29.38 ^G,H^ ± 0.65	43.66 ± 0.25	0.28 ± 0.01

Where: BAI—sum of Cad + Put + Tyr + Him; good quality meat: BAI < 5 mg·kg^−1^, acceptable meat: BAI 5–20 mg·kg^−1^, poor quality meat: BAI 20–50 mg·kg^−1^, spoiled meat: BAI > 50 mg·kg^−1^. Different superscript letters (A–J) in the same column indicate a significant difference (*p* < 0.05) between meat samples tested during 96 h of storage.

## Data Availability

The original contributions presented in the study are included in the article/Supplementary Materials, further inquiries can be directed to the corresponding author.

## Supplementary Materials

The following supporting information can be downloaded at https://www.mdpi.com/article/10.3390/foods13070985/s1, Table S1. Results of the microbial counts (average value ± standard deviation) [log_10_CFU·g^−1^] in meat samples after 48 and 96 h of storage at 4 °C; Table S2. Results of the total biogenic amines (TBAs) and TBARS contents (average value ± standard deviation) [mg·kg^−1^] in meat samples during storage at 4 °C.
