# Supplementary material for: Determination of Biogenic Amine Level Variations upon Storage, in Chicken Breast Coated with Edible Protective Film
Source: Foods. 2024 Mar 23;13(7):985. doi: 10.3390/foods13070985 (PMC11011730; doi:10.3390/foods13070985)
Supplement: Supplementary file 1 [file foods-13-00985-s001.zip › foods-2890119-supplementary.pdf]

Determination of Biogenic Amine Level Variations upon Storage, in Chicken Breast Coated with Edible Protective Film

Aneta Jastrzębska [1,*], Anna Kmieciak [2], Zuzanna Gralak [1], Kamil Brzuzy [1], Jacek Nowaczyk [3], Marcin Cichosz [4], Marek P. Krzemiński [2] and Edward Szłyk [1]

[1] Department of Analytical Chemistry and Applied Spectroscopy, Faculty of Chemistry, NCU in Toruń, 87-100 Toruń, Poland; 302301@stud.umk.pl (Z.G.); 302298@stud.umk.pl (K.B.); eszlyk@umk.pl (E.S.)
[2] Department of Organic Chemistry, Faculty of Chemistry, NCU in Toruń, 87-100 Toruń, Poland; akmieciak@umk.pl (A.K.); mkrzem@umk.pl (M.P.K.)
[3] Department of Physical Chemistry and Polymer Physical Chemistry, Faculty of Chemistry, NCU in Toruń, 87-100 Toruń, Poland; janowa@umk.pl
[4] Department of Chemical Technology, Faculty of Chemistry, NCU in Toruń, 87-100 Toruń, Poland; chemik@chem.umk.pl
* Correspondence: aj@umk.pl

Supporting materials

Table S1. Results of the microbial counts (average value ± standard deviation) [$log_{10}CFU·g^{-1}$] in meat samples after 48 and 96 hours of storage at 4^0C

	**start**	**48 hours**	**96 hours**
**CM**	**7.25±0.27**	**9.45±0.24**	**10.74±0.13**
**M-EF0.0**	**-**	**8.78±0.30**	**9.81±0.17**
**M-EF0.r**	**-**	**8.07±0.14**	**9.96±0.18**

where: **CM** - control meat, **M-EF0.0** - meat samples with emulsion coating, **M-EF0.r** - meat samples with emulsion coating and RA.

Table S2. Results of the total biogenic amines (TBAs) and TBARS contents (average value ± standard deviation) [$mg·kg^{-1}$] in meat samples during storage at 4°C

	start	24 hours	48 hours	72 hours	96 hours
TBARS					
CM	0.0785 ± 0.004	0.0833 ±0.004	0.0854 ±0.006	0.1001 ±0.002	0.124 ±0.004
M-EF0.0	-	0.0708 ±0.005	0.0782 ±0.001	0.0862 ±0.002	0.0957 ±0.006
M-EF0.r	-	0.0781 ±0.008	0.0767 ±0.003	0.0811 ±0.003	0.0904 ±0.005
SBAs					
CM	40.78 ±1.87	45.01 ±1.87	62.79 ±0.73	71.30 ±0.23	121.02 ±0.34
M-EF0.0	-	42.79 ±0.36	60.00 ±0.32	71.01 ±0.28	112.68 ±0.78
M-EF0.r	-	40.39 ±0.42	46.68 ±0.26	65.88 ±0.51	103.92 ±0.40

where: **CM** - control meat, **M-EF0.0** - meat samples with emulsion coating, **M-EF0.r** - meat samples with emulsion coating and RA; TBARS - 2- thiobarbituric acid reactive substances index; SBAs - calculated sum of all BAs
